# SERPINE1 (PAI‐1) Regulation in Mechanotransduction‐Associated Cellular Senescence

**DOI:** 10.1002/jcb.70123

**Published:** 2026-09-20

**Authors:** Intan Chairun Nisa, Pirawan Chantachotikul, Kei Takahata, Arif Nur Muhammad Ansori, Shinji Deguchi

**Affiliations:** ^1^ Division of Bioengineering, Graduate School of Engineering Science University of Osaka Toyonaka Japan; ^2^ Biotechnology Study Program Department of Applied Science, Faculty of Mathematics and Natural Sciences, Universitas Negeri Malang Malang Indonesia; ^3^ Postgraduate School, Universitas Airlangga Surabaya Indonesia; ^4^ Graduate School of Medical Sciences Kumamoto University Kumamoto Japan; ^5^ Global Center for Medical Engineering and Informatics, University of Osaka Suita Japan; ^6^ R^3^ Institute for Newly‐Emerging Science Design, University of Osaka Suita Japan

**Keywords:** cellular senescence, extracellular matrix, mechanotransduction, PAI‐1, SERPINE1

## Abstract

Cellular senescence is a complex biological process characterized by irreversible cell‐cycle arrest and acquisition of the senescence‐associated secretory phenotype (SASP). Beyond biochemical stress, increasing evidence indicates that mechanical cues from the extracellular microenvironment contribute to the regulation of senescence; however, the molecular mechanisms linking mechanotransduction to senescent phenotypes remain incompletely understood. SERPINE1 (plasminogen activator inhibitor‐1, PAI‐1) has emerged as a key mechanosensitive effector induced downstream of mechanotransduction signaling pathways. In this review, we discuss SERPINE1 as both a hallmark of cellular senescence and a functional mediator linking mechanotransduction to senescence‐associated processes. Once induced, SERPINE1 contributes to cell‐cycle arrest and extracellular matrix (ECM) remodeling by inhibiting plasmin‐dependent proteolysis. The resulting ECM accumulation and matrix stiffening further enhance integrin‐dependent mechanotransduction, promoting further SERPINE1 expression and establishing a self‐reinforcing mechanobiological feedback loop. SERPINE1 also contributes to senescence‐associated inflammatory signaling, which may further support the maintenance of the senescent phenotype. Together, these findings identify SERPINE1 as a mechanistic link between mechanotransduction, ECM remodeling, and cellular senescence. Pharmacological studies using PAI‐1 inhibitors further support the biological importance of this pathway and suggest that SERPINE1 may represent a potential therapeutic target for senescence‐associated fibrotic diseases.

## 1 Introduction

Living cells continuously perceive and adapt to the physical properties of their microenvironment. Extracellular matrix (ECM) stiffness, tensile strain, and shear stress act as physical cues that are converted into intracellular biochemical signals regulating gene expression and cellular behavior; this process is known as mechanotransduction [1, 2]. These biophysical signaling pathways dictate fundamental cellular behaviors, including proliferation, differentiation, migration, and survival. Conversely, the dysregulation of these pathways is increasingly implicated in pathological tissue remodeling and age‐related phenotypes, such as tissue fibrosis, vascular stiffening, and chronic degenerative disorders [2, 3, 4].

Among the molecular targets modulated by mechanotransduction, SERPINE1 (plasminogen activator inhibitor‐1, PAI‐1) has emerged as a prominent mechanosensitive gene whose expression is tightly coupled to alterations in microenvironmental mechanics [3, 5]. Beyond functioning as a mechanosensitive target, SERPINE1 also contributes to extracellular matrix remodeling. It primarily inhibits tissue‐type (tPA) and urokinase‐type (uPA) plasminogen activators, which otherwise catalyze the conversion of plasminogen to active plasmin [6, 7]. By inhibiting tPA and uPA, SERPINE1 reduces plasmin generation and limits plasmin‐dependent ECM degradation, thereby favoring ECM persistence and accumulation. These changes may contribute to matrix stiffening, depending on ECM composition, crosslinking, organization, and cellular tension. This remodeling of the cell–matrix interface alters the mechanical microenvironment and thereby reinforces mechanotransduction signaling [5, 8].

In addition, SERPINE1 is a well‐established hallmark of cellular senescence. Senescence is a state of irreversible growth arrest that limits the proliferation of damaged cells and contributes to aging and age‐related diseases [9, 10, 11, 12]. SERPINE1 expression is elevated in senescent cells and is regarded as part of the senescence‐associated secretory phenotype (SASP) [13, 14, 15], supporting its role as a molecular marker of senescence. Accumulating evidence suggests that SERPINE1 contributes to the establishment and maintenance of senescence through mechanisms involving cell‐cycle regulation, pro‐inflammatory signaling, and particularly ECM remodeling [8, 14, 16, 17]. These observations suggest a bidirectional relationship between mechanotransduction and SERPINE1 in the context of cellular senescence. In this review, we integrate current evidence into a cohesive mechanistic framework that maps out mechanosensitive SERPINE1 induction, its downstream role in cellular senescence, and the reciprocal feedback loop through which SERPINE1‐driven ECM remodeling sustains mechanotransduction signaling. Evidence discussed in this review is drawn primarily from fibroblast and epithelial cell contexts, where substantial mechanistic evidence for SERPINE1‐associated senescence is currently available, while selected examples from other mechanically responsive tissues are included to illustrate broader disease relevance.

## 2 Mechanotransduction‐Mediated Induction of SERPINE1

### 2.1 Mechanical Cues and Transcription Activation

Mechanical cues from the extracellular environment are important regulators of mechanosensitive gene expression. Cells continuously sense and adapt to ECM stiffness, tensile forces, and actomyosin‐generated tension through mechanosensors including integrins, focal adhesions, and the actomyosin cytoskeleton [18, 19, 20]. These mechanical inputs are transmitted to the nucleus via connected biochemical pathways and direct structural connections between the cytoplasm and the nucleus, ultimately leading to changes in the expression of mechanosensitive genes [19, 20, 21].

Within this regulatory landscape, SERPINE1 has emerged as a prominent mechanosensitive gene that is strongly upregulated by mechanical cues [5]. Increasing substrate stiffness induces SERPINE1 in fibroblast models and has been associated with increased SERPINE1 expression in selected pathological contexts [22]. This transcriptional control relies on a network of mechanosensitive transcription factors (TFs) and regulatory proteins that bind to the SERPINE1 promoter either directly, through promoter‐associated transcription factors, or indirectly through cooperating signaling networks. Table 1 summarizes the principal transcriptional regulators of SERPINE1 downstream of mechanotransduction signaling pathways.

**Table 1:** Mechanotransduction‐associated transcriptional regulators of SERPINE1 expression.

	Category	TFs	Co‐regulator(s)	Signaling/Regulation	References
1	Core mechanotransduction‐associated TFs	TEAD	YAP/TAZ	ECM stiffness → RhoA/ROCK → actomyosin tension → YAP/TAZ–TEAD	[5, 23]
2	Core mechanotransduction‐associated TFs	SMAD2/3/4	p300/CBP	TGF‐β → TGFβR → pSMAD2/3 → SMAD2/3‐SMAD4	[24, 25, 26]
3	Context‐dependent convergence TFs	AP‐1 (c‐Jun/c‐Fos)	p300/CBP	Integrin/FAK/Src → MAPK (ERK/JNK/p38) → AP‐1 activation	[27, 28]
4	Context‐dependent convergence TFs	NF‐κB p65/p50	p300/CBP	Mechano‐inflammation → IKK/IκB → NF‐κB p65	[29, 30]
5	Context‐dependent convergence TFs	HIF‐1α/HIF‐1β	p300/CBP	Hypoxia/ROS → HIF‐1α	[31, 32]
6	Context‐dependent convergence TFs	p53	SMAD2/3, p300/CBP	Nuclear strain → DNA damage/ROS → ATM/ATR → p53 stabilization	[26, 33]

Abbreviations: AP‐1, activator protein‐1; ATM, ataxia telangiectasia mutated; ATR, ataxia telangiectasia and Rad3‐related; CBP, CREB‐binding protein; ECM, extracellular matrix; ERK, extracellular signal‐regulated kinase; FAK, focal adhesion kinase; HIF‐1α/HIF‐1β, hypoxia‐inducible factor‐1α/hypoxia‐inducible factor‐1β; IKK, IκB kinase; IκB, inhibitor of κB; JNK, c‐Jun N‐terminal kinase; MAPK, mitogen‐activated protein kinase; NF‐κB, nuclear factor kappa B; pSMAD2/3, phosphorylated SMAD2/3; RhoA, Ras homolog family member A; ROCK, Rho‐associated protein kinase; ROS, reactive oxygen species; TGF‐β, transforming growth factor beta; TGFβR, TGF‐β receptor; TF, transcription factor; TAZ, transcriptional coactivator with PDZ‐binding motif; TEAD, TEA domain transcription factor; YAP, yes‐associated protein.

### 2.2 Core Mechanosensitive Signaling Axes

The transcriptional regulators listed in Table 1 can be broadly classified into core mechanotransduction TFs and convergent TFs. Core mechanotransduction TFs link mechanical cues to SERPINE1 transcription, whereas convergent TFs integrate mechanotransduction with inflammatory, metabolic, or stress‐responsive signaling. The YAP/TAZ–TEAD and TGF‐β/SMAD pathways represent the two core mechanotransduction pathways discussed below.

#### 2.2.1 The YAP/TAZ–TEAD Axis

**Figure 1:**
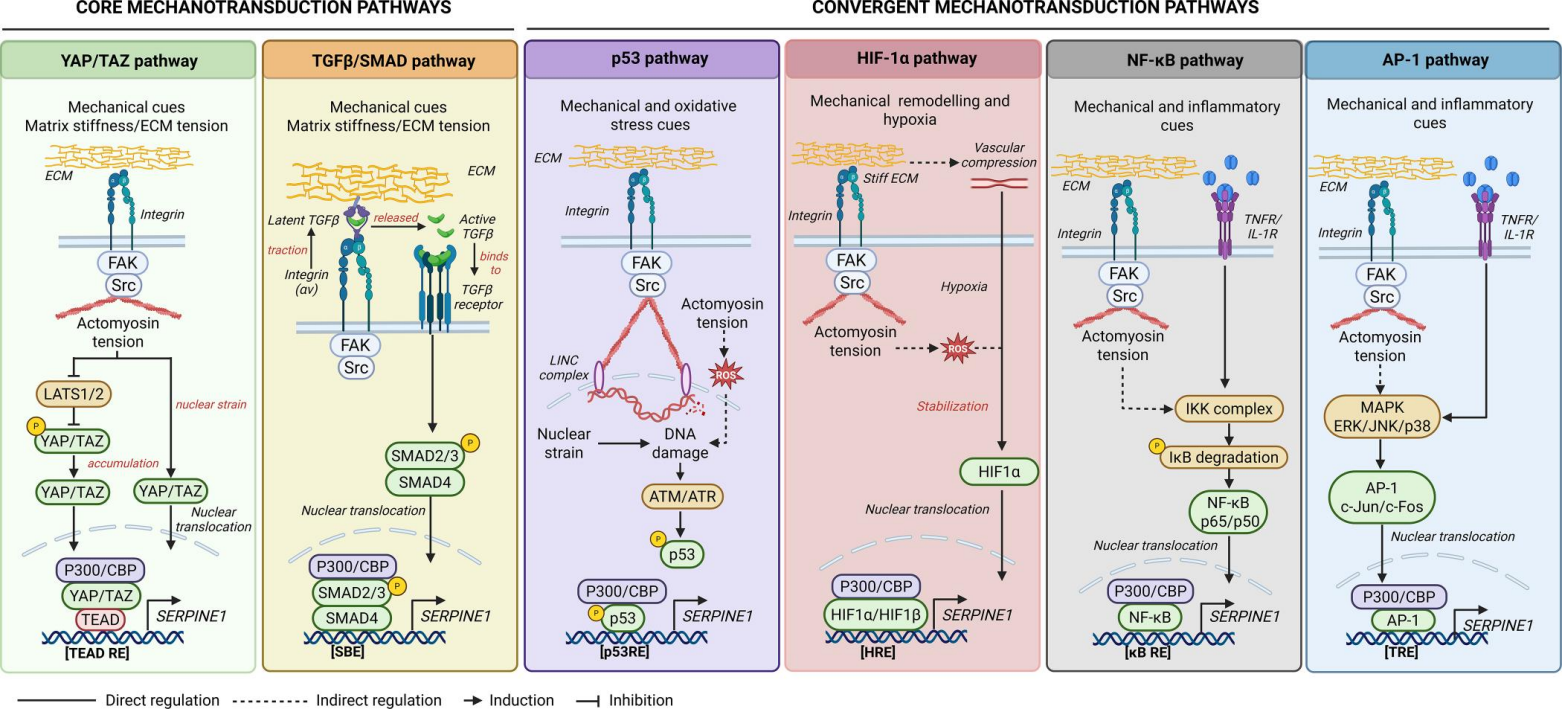
Mechanotransduction‐associated transcriptional regulation of SERPINE1. Mechanical cues from the extracellular microenvironment are sensed through integrin‐mediated adhesion and transmitted via mechanotransduction pathways, including TGF‐β/SMAD, RhoA/ROCK, and MAPK signaling. These signals converge in the nucleus to regulate SERPINE1 transcription through two conceptual groups. The core mechanotransduction‐associated axes are YAP/TAZ–TEAD and TGF‐β/SMAD, which provide well‐supported mechanistic links between mechanical cues and SERPINE1 transcription. In contrast, AP‐1, NF‐κB, HIF‐1α, and p53 are shown as context‐dependent convergent regulators that integrate mechanotransduction with inflammatory, hypoxic/oxidative, or genotoxic stress signaling. Solid arrows indicate direct interactions; dashed arrows indicate indirect or multi‐step signaling; and the thick bold arrow indicates the transcription start site. ECM, extracellular matrix; FAK, focal adhesion kinase; Src, proto‐oncogene tyrosine‐protein kinase Src; RhoA, Ras homolog family member A; ROCK, Rho‐associated protein kinase; YAP/TAZ, yes‐associated protein/transcriptional coactivator with PDZ‐binding motif; TEAD, TEA domain transcription factor; MAPK, mitogen‐activated protein kinase; AP‐1, activator protein‐1; NF‐κB, nuclear factor kappa B; HIF‐1α, hypoxia‐inducible factor 1‐alpha; TGF‐β, transforming growth factor beta.

The YAP/TAZ–TEAD axis provides a well‐supported mechanistic link between matrix stiffness and SERPINE1 expression. On stiff matrices, integrin clustering and focal adhesion maturation activate focal adhesion kinase (FAK)–Src and Ras homolog family member A (RhoA)–Rho‐associated protein kinase (ROCK) signaling, thereby increasing actomyosin contractility (Figure 1) [23, 34]. Matrix stiffness promotes RhoA–ROCK‐dependent actomyosin tension, which then leads to YAP/TAZ nuclear accumulation [5].

In some cellular systems, actomyosin tension modulates large tumor suppressor kinase 1/2 (LATS1/2)‐dependent inhibitory phosphorylation of YAP/TAZ, and reduced phosphorylation favors their nuclear accumulation and transcriptional activity [35]. However, rigidity‐dependent YAP/TAZ activation can also occur independently of the canonical Hippo–LATS cascade through Rho GTPase‐dependent actomyosin tension [23]. In addition, mechanical forces transmitted from focal adhesions to the nucleus can flatten the nucleus and stretch nuclear pore complexes, thereby increasing YAP nuclear import [36]. Because YAP and TAZ lack intrinsic DNA‐binding domains, they associate with TEAD family transcription factors to regulate mechanosensitive transcriptional programs [37, 38]. In lung fibroblasts, increasing matrix stiffness promotes YAP/TAZ nuclear accumulation and increases SERPINE1/PAI‐1 expression, whereas YAP/TAZ knockdown attenuates stiffness‐induced SERPINE1 expression, supporting SERPINE1 as a YAP/TAZ‐dependent mechanosensitive target [5].

#### 2.2.2 The TGF‐β/SMAD Pathway

The TGF‐β/SMAD signaling pathway provides a well‐supported mechanotransduction‐associated route linking extracellular mechanical cues to SERPINE1 induction [39, 40, 41]. On a sufficiently resistant ECM, actomyosin‐generated traction is transmitted through selected αv‐containing integrins that recognize the arginine‐glycine‐aspartate (RGD) motif within the latency‐associated peptide (LAP). Because latent transforming growth factor beta‐binding protein 1 (LTBP‐1) anchors the latent complex to the ECM, integrin‐mediated pulling generates opposing forces across the complex and mechanically deforms LAP, a structural component of the latent TGF‐β1 complex that maintains TGF‐β1 latency [42, 43]. This deformation exposes or releases biologically active TGF‐β1 without requiring proteolytic cleavage of the latent complex (Figure 1) [24, 42, 43].

Active TGF‐β1 subsequently binds to the type II TGF‐β receptor, which recruits and activates the type I receptor. The activated type I receptor phosphorylates SMAD2 and SMAD3, which then associate with SMAD4 and accumulate in the nucleus [44]. Within the nucleus, SMAD3/SMAD4 complexes bind directly to CAGA‐containing SMAD‐binding elements in the human SERPINE1 promoter and stimulate SERPINE1 transcription [25, 45]. SMAD proteins can also interact with the transcriptional coactivators p300 and CREB‐binding protein (CBP), thereby enhancing SMAD‐dependent transcriptional activity [46, 47]. Thus, cell‐generated contractile forces and ECM resistance can regulate SERPINE1 expression through mechanical activation of matrix‐bound latent TGF‐β1 followed by canonical SMAD signaling.

### 2.3 Convergence of Stress and Inflammatory Signaling

In contrast to the core mechanotransduction TFs, context‐dependent convergence TFs such as activator protein‐1 (AP‐1), nuclear factor kappa B (NF‐κB), p53, and hypoxia‐inducible factor 1‐alpha (HIF‐1α) integrate mechanical cues with inflammatory, metabolic, and stress‐related signaling. The following pathways are not proposed as universal or primary mechanosensors. Rather, they represent stress‐responsive pathways that may converge with mechanotransduction to enhance or sustain SERPINE1 expression in specific cellular or pathological contexts. Moreover, the contribution of these TFs to SERPINE1 expression induced by mechanical cues has yet to be fully elucidated. Therefore, the complete sequence from mechanical stimulation to SERPINE1 transcription has not been demonstrated within a single cell type or experimental system.

#### 2.3.1 AP‐1 and MAPK Signaling

Activator protein‐1 (AP‐1) may function as a context‐dependent point of convergence between mechanical, inflammatory, and profibrotic signaling rather than as a primary mechanosensor. In fibroblast models, mechanical stretch can activate mitogen‐activated protein kinase (MAPK) pathways in an integrin‐ and matrix‐dependent manner. Static biaxial stretch activates extracellular signal‐regulated kinase (ERK) and c‐Jun N‐terminal kinase (JNK) in cardiac fibroblasts, whereas mechanical loading of human periodontal ligament fibroblasts activates ERK/JNK signaling and AP‐1‐dependent transcriptional responses [28, 48]. Depending on the cell type and loading conditions, p38 MAPK and increased expression of the AP‐1 components c‐Fos and c‐Jun may also accompany the mechanical response [49].

Inflammatory signals can converge on the same MAPK–AP‐1 machinery. In human fibroblasts, tumor necrosis factor alpha (TNF‐α) induces sustained JNK activation and phosphorylation‐dependent activation of c‐Jun, whereas interleukin‐1 (IL‐1) activates ERK, JNK, and p38 and induces c‐Fos expression through signaling that is influenced by focal‐adhesion organization [50, 51]. Separate promoter studies provide evidence that AP‐1 can directly contribute to SERPINE1 transcription under specific biochemical conditions. In fibroblasts, the fibrin degradation product D‐dimer increased binding of a c‐Fos/JunD complex to a conserved AP‐1‐like element within the proximal SERPINE1 promoter. Mutation of this element reduced basal, c‐Fos‐induced, and D‐dimer‐induced promoter activity, demonstrating a functional role for this site in SERPINE1 transcription [27]. Collectively, these observations support the possibility that mechanical and inflammatory inputs coexist and converge on AP‐1 signaling within chronically remodeled tissue microenvironments.

#### 2.3.2 NF‐κB and Inflammatory Signaling

NF‐κB is best regarded as a context‐dependent inflammatory and stress‐responsive regulator that may converge with mechanotransduction to influence SERPINE1 expression, rather than as a primary mechanosensor. In resting cells, canonical NF‐κB complexes, commonly composed of RelA/p65 and p50, are retained in the cytoplasm through their association with inhibitor of κB (IκB) proteins. Pro‐inflammatory cytokines such as TNF‐α and interleukin‐1 beta (IL‐1β) activate the IκB kinase (IKK) complex, leading to IκBα phosphorylation, ubiquitination, and proteasomal degradation. The released NF‐κB complex subsequently translocates into the nucleus and regulates inflammatory and stress‐responsive transcriptional programs [52, 53].

Mechanical stimulation can also activate NF‐κB in selected cellular contexts, although the upstream mechanism is not universal. In human fibroblasts, cyclic stretch activated stretch‐sensitive Ca^2+^ entry, increased reactive oxygen species (ROS) production, stimulated IKK activity and IκB phosphorylation, and promoted nuclear translocation of RelA/p65 [30]. However, the downstream target examined in that study was cyclooxygenase‐2 rather than SERPINE1. Thus, this evidence establishes a mechanical route to NF‐κB activation but does not directly demonstrate mechanical induction of SERPINE1 through NF‐κB.

Independent inflammatory studies provide more direct evidence that NF‐κB can regulate SERPINE1 transcription. In the human SERPINE1 locus, TNF‐α activates a distal enhancer located approximately 15 kb upstream of the transcription start site. This enhancer contains a functional κB‐binding element that binds the NF‐κB subunits p50 and p65, whereas inhibition of NF‐κB prevents TNF‐α‐dependent enhancer activation [29]. In mouse embryonic fibroblasts, TNF‐α and lipopolysaccharide increased Pai‐1 expression through a proximal κB‐responsive element. NF‐κB‐dependent regulation was supported by p50/p65 binding, chromatin immunoprecipitation, promoter‐reporter assays, site‐directed mutation of the κB element, pharmacological NF‐κB inhibition, and p65 knockdown [54]. These findings indicate that inflammatory NF‐κB signaling can directly regulate SERPINE1, although the location and organization of the relevant regulatory elements may differ among species and cellular contexts.

#### 2.3.3 HIF‐1α and Hypoxic Stress

Hypoxia‐inducible factor‐1α (HIF‐1α) is best regarded as a context‐dependent hypoxic and oxidative‐stress regulator that may converge with mechanotransduction to promote SERPINE1 expression, rather than as a primary mechanosensor. Under normoxic conditions, HIF‐1α is hydroxylated at specific proline residues, recognized by the von Hippel–Lindau protein, ubiquitinated, and rapidly degraded through the proteasome [55, 56]. Under hypoxic conditions, oxygen‐dependent hydroxylation is reduced, allowing HIF‐1α to accumulate and become transcriptionally active. Hypoxia‐associated mitochondrial reactive oxygen species can also contribute to HIF‐1α stabilization in selected cellular contexts [57].

Stabilized HIF‐1α accumulates in the nucleus and heterodimerizes with the constitutively expressed HIF‐1β subunit. The resulting HIF‐1 complex binds hypoxia‐response elements within target‐gene regulatory regions and recruits transcriptional coactivators, including p300 and CBP, to enhance hypoxia‐responsive transcription [58]. Direct promoter studies have demonstrated that mild hypoxia induces SERPINE1 transcription through functional HIF‐1‐binding hypoxia‐response elements in the rat SERPINE1 promoter; mutation of these elements abolishes the hypoxic response [32].

Mechanically remodeled tissues may generate secondary hypoxic microenvironments through several tissue‐level processes. In highly fibrotic or desmoplastic tissues, excessive accumulation of collagen, hyaluronan, and other extracellular matrix components contributes to the development and transmission of solid stress. These compressive stresses can narrow or collapse microvessels, reduce vascular perfusion, and limit oxygen delivery, thereby promoting tissue hypoxia [59, 60].

More recently, hypoxia‐induced ROS were shown to promote HIF‐1α accumulation and HIF‐1α‐dependent SERPINE1 promoter activity in human glioblastoma cells, supporting direct HIF‐1α‐mediated SERPINE1 regulation in a human pathological context [31]. These tissue‐level changes could therefore reinforce SERPINE1 expression through HIF‐1α signaling. However, direct continuity from ECM stiffness or actomyosin tension to HIF‐1α‐dependent SERPINE1 transcription has not been established as a universal cell‐autonomous mechanism.

#### 2.3.4 p53 and Nuclear Mechanical Stress

p53 is best regarded as a context‐dependent mechanical–genotoxic convergence factor rather than as a direct mechanosensor. Extracellular and intracellular forces can be transmitted from the actin cytoskeleton to the nuclear envelope through the linker of nucleoskeleton and cytoskeleton (LINC) complex and provide a physical connection between cytoskeletal filaments, nuclear‐envelope proteins, and the nuclear lamina [61]. This nucleocytoskeletal coupling allows mechanical forces to influence nuclear shape and organization.

Excessive mechanical strain deforms the nuclear envelope and may induce transient nuclear envelope rupture, leading to DNA damage and activation of the ataxia telangiectasia mutated (ATM)/ataxia telangiectasia and Rad3‐related (ATR) signaling pathway [62, 63, 64]. DNA‐damage signaling through ATM/ATR‐associated pathways can stabilize and activate p53. Activated p53 can directly regulate SERPINE1 transcription. Wild‐type p53 binds and activates the human SERPINE1 promoter, whereas oncogenic p53 mutants lose this activity [65]. In addition, genotoxic stress induced SERPINE1 in a p53‐dependent manner through a functional p53‐response element (p53RE) located in the proximal SERPINE1 promoter; mutation of this element abolished p53‐dependent promoter induction [33].

p53 can also cooperate with TGF‐β/SMAD signaling in SERPINE1 regulation. In human cell models, TGF‐β promoted formation of a p53–Smad2/3 complex at the SERPINE1 promoter [26]. This finding supports transcriptional convergence between p53 and SMAD signaling, but it does not by itself demonstrate that matrix stiffness activates the complete LINC–DNA damage–p53–SMAD–SERPINE1 sequence.

## 3 Roles of SERPINE1 in Senescence

SERPINE1 contributes to the senescent phenotype through both extracellular matrix remodeling and intracellular cell‐cycle regulation. In the extracellular compartment, secreted SERPINE1 inhibits tPA and uPA, thereby reducing plasmin generation. Reduced plasmin activity limits direct extracellular proteolysis and the activation of selected matrix metalloproteinase (MMP) zymogens (pro‐MMPs), favoring ECM persistence and accumulation. These changes may contribute to matrix stiffening, although tissue stiffness also depends on ECM composition, crosslinking, organization, and cellular tension [8]. This remodeling of the extracellular microenvironment alters cell–matrix interactions and enhances the mechanical signals transmitted through integrins.

These extracellular mechanical signals propagate through focal adhesions to promote structural remodeling of the actomyosin cytoskeleton. Proteomic analyses of senescent human fibroblasts have shown that cellular senescence is accompanied by reinforcement of the stress fiber network, characterized by increased expression of stress fiber‐associated proteins [66]. In that analysis, SERPINE1 was identified as one of the stress fiber‐associated proteins and was upregulated during senescence. These findings suggest that SERPINE1 is associated with the stress fiber network in senescent cells and may be linked to reinforced stress fibers and altered cytoskeletal organization, although whether SERPINE1 directly drives these cytoskeletal changes remains unclear. Because stress fiber formation is enhanced on stiff ECM, these observations further suggest that SERPINE1‐associated cytoskeletal remodeling may cooperate with ECM stiffening to stabilize the senescent phenotype [2, 67, 68]. This sustained mechanical stress is also associated with morphological remodeling, reduced collagen turnover, and persistent intracellular ROS accumulation, which may contribute to the maintenance of the senescent phenotype.

**Figure 2:**
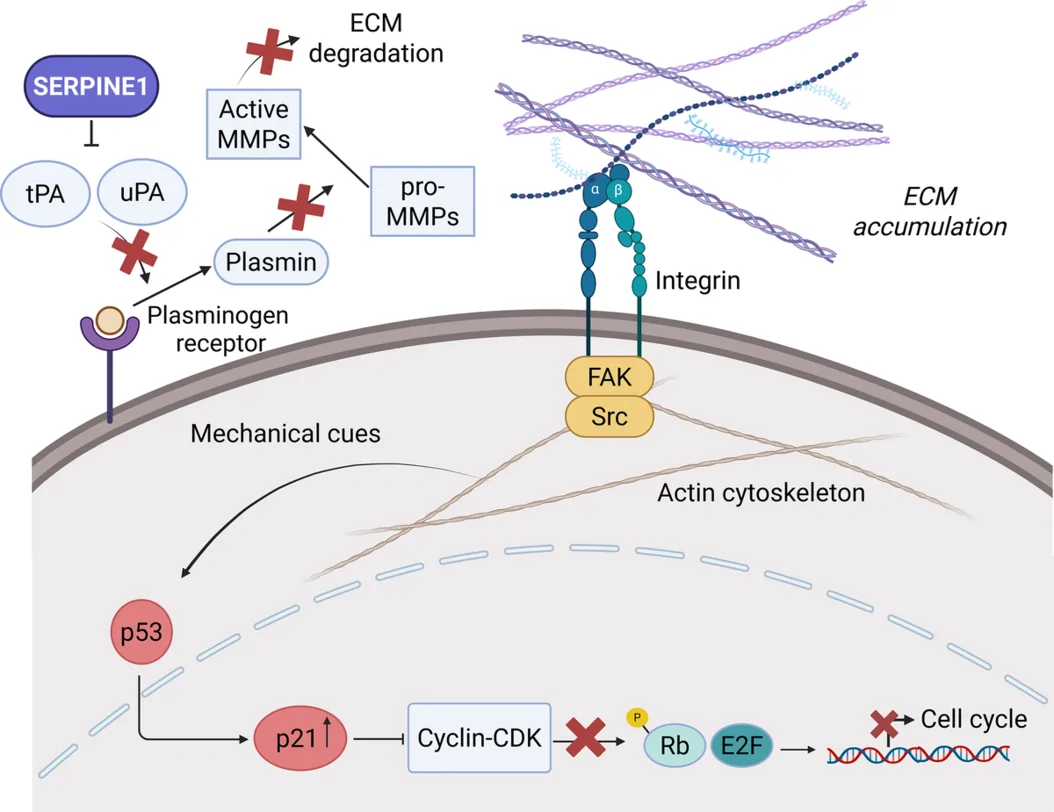
SERPINE1‐mediated control of ECM degradation and mechanotransduction‐associated cell‐cycle arrest. SERPINE1 inhibits tPA and uPA, thereby reducing plasmin generation. Plasmin contributes to pro‐MMP activation, thereby promoting matrix degradation. Consequently, SERPINE1‐mediated suppression of plasmin activity may limit ECM degradation and favor ECM accumulation. The accumulated and stiffened ECM is sensed by integrins, and the resulting mechanical signals are transmitted intracellularly through FAK, Src, and cytoskeletal remodeling, generating mechanical stress signals. These mechanical inputs may converge on the p53–p21–Rb pathway, leading to inhibition of cyclin–CDK activity, maintenance of Rb‐mediated E2F repression, and cell‐cycle arrest.

Beyond its effects on extracellular matrix remodeling, SERPINE1 also contributes to the senescent phenotype through intracellular mechanisms [16, 17, 69]. At the intracellular level, SERPINE1 may promote cell‐cycle arrest through the p53–p21–Rb pathway. In contrast, its relationship with the p16^INK4α^ pathway appears to be context‐dependent; rather than functioning as a direct upstream initiator, SERPINE1 accumulation typically correlates with p16^INK4α^ upregulation during late‐stage senescence reinforcement or tissue aging [70]. Elevated SERPINE1 may promote activation of the p53‐dependent pathway, resulting in increased p21 expression, inhibition of cyclin‐dependent kinases (CDKs), maintenance of the retinoblastoma (Rb) protein in a hypophosphorylated state, and suppression of E2F‐dependent transcription (Figure 2) [17, 69, 71].

Mechanical stress, matrix stiffening, and sustained ROS accumulation have also been associated with increased p16^INK4α^ expression. Once expressed, p16^INK4α^ inhibits CDK4 and CDK6, preventing Rb phosphorylation and reinforcing stable cell‐cycle arrest. The coordinated activation of the p53–p21–Rb and p16^INK4α^ pathways suggests that SERPINE1 may contribute to growth arrest even when p53 function is compromised, although direct evidence from fully p53‐null settings remains limited. In addition, as a component of the SASP, secreted SERPINE1 may act through autocrine and paracrine mechanisms to promote inflammatory signaling and contribute to the maintenance of the senescent phenotype [11, 13].

## 4 Proposed SERPINE1‐Driven Mechanobiological Feedback Loop in Cellular Senescence

**Figure 3:**
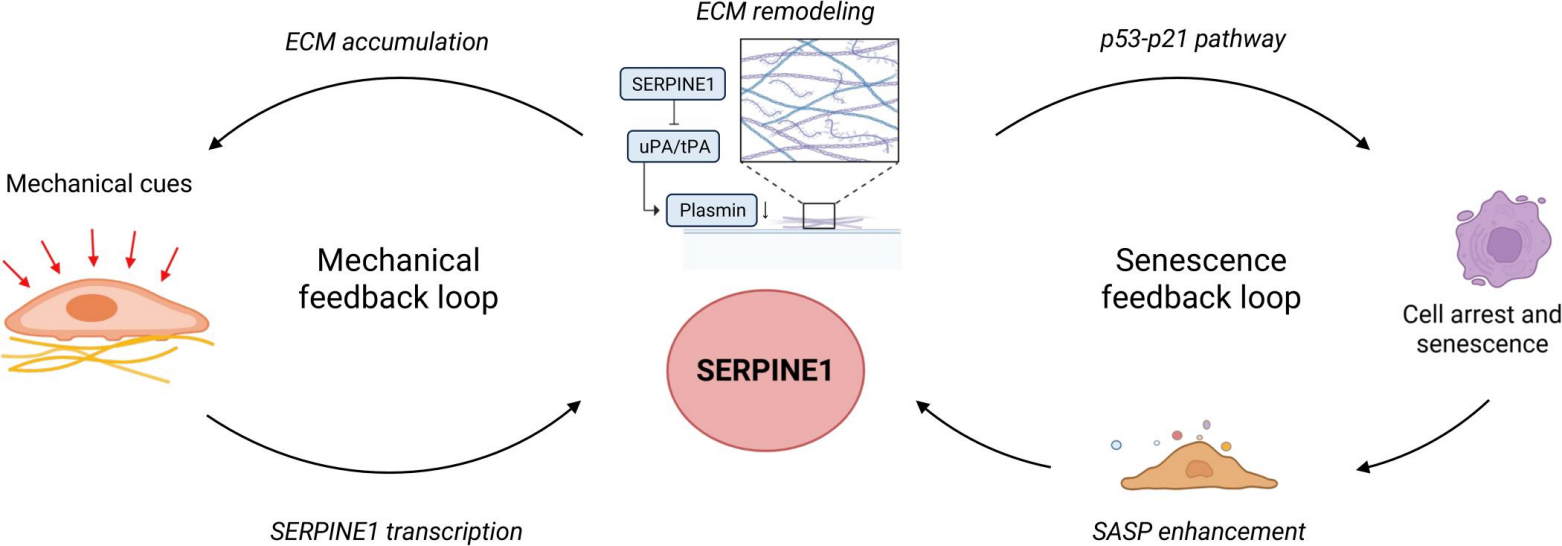
Proposed mechanical and senescence‐associated feedback loops linking ECM stiffness, SERPINE1, and cellular senescence. Mechanical cues, including ECM stiffness, shear stress, and stretch, activate mechanotransduction pathways involving integrins, FAK, RhoA/ROCK, YAP/TAZ–TEAD, and SMAD2/3, while context‐dependent pathways such as AP‐1 and p53 may additionally converge on SERPINE1 regulation. Increased SERPINE1 inhibits tPA/uPA‐mediated plasmin generation, thereby reducing ECM degradation and promoting ECM accumulation and matrix stiffening. The resulting stiffened ECM further enhances mechanotransduction signaling, forming a mechanical feedback loop. In parallel, mechanical stress and SERPINE1‐associated signaling may contribute to p53–p21–Rb‐mediated cell‐cycle arrest and cellular senescence. Senescent cells produce SASP factors, including IL‐6, IL‐8, IL‐1, and TGF‐β, which may further promote SERPINE1 expression and ECM remodeling, establishing a senescence‐associated feedback loop.

The role of SERPINE1 in mechanotransduction‐associated senescence can be understood through two interconnected feedback loops: a mechanical feedback loop and a senescence‐associated feedback loop. In the mechanical feedback loop, SERPINE1 functions both as a downstream target of mechanosensitive signaling and as an extracellular regulator that remodels the mechanical microenvironment. Secreted SERPINE1 inhibits tPA/uPA‐mediated plasmin generation, thereby reducing plasmin‐dependent activation of matrix‐degrading MMPs. This reduction in pericellular proteolysis promotes ECM accumulation and matrix stiffening [5, 8]. The stiffened ECM is sensed by integrins and focal adhesions, activating FAK/Src, RhoA/ROCK, actomyosin contractility, and downstream transcriptional regulators. These signaling pathways support a proposed self‐reinforcing mechanobiological feedback model involving SERPINE1 in which ECM remodeling sustains mechanotransduction signaling and SERPINE1 expression (Figure 3).

In parallel, SERPINE1 also participates in a senescence‐associated feedback loop that is not exclusively initiated by mechanical cues. Classical senescence triggers, including DNA damage, oxidative stress, oncogenic signaling, inflammation, and TGF‐β stimulation, can induce SERPINE1 expression through p53‐, SMAD‐, NF‐κB‐, and AP‐1‐dependent pathways [14, 39, 72]. Once upregulated, SERPINE1 may reinforce cell‐cycle arrest through the p53–p21–Rb pathway and, in some contexts, cooperate with p16^INK4α^‐dependent growth arrest [17, 72]. Senescent cells then secrete SASP factors, including interleukin‐6 (IL‐6), interleukin‐8 (IL‐8), TGF‐β, and SERPINE1 itself. These SASP mediators act in an autocrine and paracrine manner to sustain inflammatory signaling, activate NF‐κB/signal transducer and activator of transcription 3 (STAT3) and TGF‐β/SMAD pathways, and promote further SERPINE1 transcription [15, 39, 73]. Therefore, SERPINE1 may contribute to the maintenance of senescence not only by altering ECM mechanics but also by reinforcing SASP‐driven inflammatory and profibrotic signaling.

Importantly, these two feedback loops are interconnected rather than independent processes. Mechanical stiffening can promote senescence through nuclear deformation, ROS accumulation, DNA damage signaling, and activation of the p53–p21 pathway. Conversely, senescent cells remodel the surrounding ECM through SASP‐associated secretion of SERPINE1, TGF‐β, inflammatory cytokines, and matrix‐remodeling enzymes. Together, these interconnected feedback loops suggest that SERPINE1 contributes to the maintenance of the senescent phenotype [14, 74, 75].

## 5 Implications of SERPINE1 in Senescence and Disease

**Figure 4:**
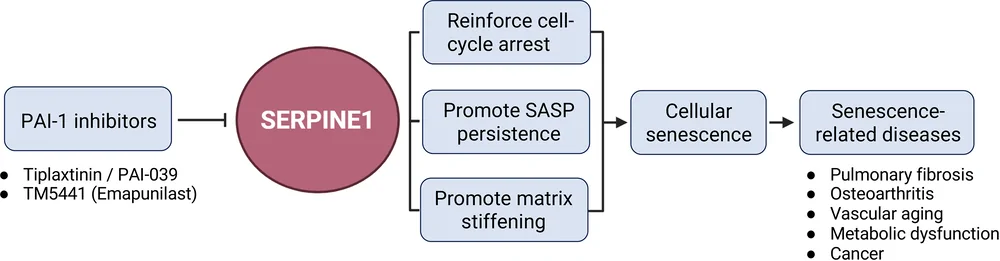
Summary of the implications of SERPINE1 in cellular senescence and senescence‐related diseases. SERPINE1 contributes to the persistence of cellular senescence through multiple mechanisms, including reinforcement of cell‐cycle arrest, maintenance of SASP signaling, and promotion of ECM remodeling. Together, these processes may contribute to the maintenance of the senescent phenotype and to senescence‐related diseases, particularly fibrotic and age‐associated tissue dysfunction. Pharmacological inhibition of SERPINE1 may interfere with these processes, highlighting the biologically relevance of SERPINE1 and its potential as a therapeutic target in senescence‐associated pathology.

SERPINE1 is increasingly recognized as more than a senescence‐associated marker. Accumulating evidence suggests that SERPINE1 contributes to the persistence of senescence‐associated tissue dysfunction by reinforcing cell‐cycle arrest, sustaining SASP‐related signaling, and promoting extracellular matrix remodeling (Figure 4). Because SERPINE1 accumulates both locally within damaged tissues and systemically in the circulation, it serves as a useful biomarker of senescent cell burden in vivo [13, 14, 62]. Its elevated expression across numerous age‐related and fibrotic disorders further suggests that SERPINE1 may serve as an important molecular link between chronic cellular stress and tissue dysfunction [3, 62].

In fibroblast‐associated pathologies, SERPINE1 contributes to disease progression by suppressing matrix proteolysis, promoting ECM accumulation, and increasing tissue stiffness. These processes contribute to fibrotic remodeling in disorders including idiopathic pulmonary fibrosis (IPF), vascular aging, cancer [16, 71, 76], obesity, and type 2 diabetes [13, 62]. In IPF fibroblasts, stiffness‐mediated SERPINE1 induction has been reported together with other mechanosensitive profibrotic genes, contributing to a self‐reinforcing fibrotic microenvironment. Similarly, single‐cell transcriptomic analyses of chronic heart failure have identified activated fibroblast populations enriched in ECM–receptor interaction pathways and SERPINE1‐associated remodeling programs, supporting a link between force‐dependent SERPINE1 expression and myocardial fibrosis [5, 77].

Epithelial cell senescence represents another important context in which SERPINE1 contributes to tissue degeneration. In alveolar epithelial cells, elevated SERPINE1 has been associated with increased expression of p53, p21, and p16^INK4α^ and with cellular senescence, contributing to disruption of alveolar integrity in fibrotic lung disease. SERPINE1 may also contribute to stromal–epithelial communication. For example, fibroblast‐derived SERPINE1 disrupts epithelial vitronectin–integrin signaling, promoting focal adhesion disassembly and paracrine epithelial senescence [75].

Osteoarthritis represents another mechanobiological context in which SERPINE1 may contribute to matrix remodeling. Articular chondrocytes are highly responsive to mechanical cues. Compressive loading activates SMAD2/3 signaling and upregulates SERPINE1 in articular cartilage [78], whereas fluid shear stress directly stimulates PAI‐1 expression in human osteoarthritic chondrocytes [79]. In inflammatory or mechanically stressed joints, elevated SERPINE1 has been associated with increased expression of matrix metalloproteinases (MMP‐3, MMP‐9, and MMP‐13), which contributes to cartilage degeneration [80]. Altered mechanical properties of osteoarthritic cartilage, including regional increases in matrix stiffness, may further enhance mechanosensitive signaling and contribute to a degenerative feedback loop [81].

The broader clinical and biological significance of SERPINE1 is further supported by genetic epidemiological evidence. Individuals heterozygous for a naturally occurring null SERPINE1 allele in the Amish cohort exhibit prolonged lifespan, improved metabolic profiles, and reduced biological aging, supporting a role for SERPINE1 in age‐related systemic decline [62]. Collectively, these findings suggest that diverse age‐related diseases share common features, including ECM stiffening, persistent mechanotransduction signaling, and stable cellular senescence [67, 68]. Although these conditions affect different tissues, SERPINE1 may represent a common mechanistic link by contributing to extracellular matrix remodeling and the maintenance of a senescence‐supportive microenvironment [17, 62, 75, 82].

Importantly, the biological relevance of SERPINE1 is further supported by pharmacological studies. Several PAI‐1 inhibitors, including tiplaxtinin (PAI‐039), TM5441, TM5275, and TM5509, have been investigated in preclinical models. TM5441 attenuates vascular senescence, hypertension, cardiac hypertrophy, and periaortic fibrosis in mice while reducing p16^INK4α^ expression and preserving telomere length [83]. In cellular models, TM5441 also protects cardiomyocytes, fibroblasts, and endothelial cells from doxorubicin‐induced senescence and reduces replicative senescence in fibroblasts [84]. These findings support the biological importance of SERPINE1 in senescence‐associated pathology and suggest that SERPINE1 may represent a potential therapeutic target rather than merely a biomarker of cellular senescence.

## 6 Challenges and Future Directions

While substantial evidence supports a role for SERPINE1 as a mechanobiological mediator linking mechanotransduction to cellular senescence, several challenges remain. First, most mechanistic insights have been obtained from two‐dimensional fibroblast cultures on polyacrylamide substrates, whereas physiological validation will require three‐dimensional organoids and in vivo animal models [85]. Although these simplified systems have been instrumental in dissecting stiffness‐dependent signaling, they do not fully recapitulate the structural complexity, multicellular interactions, and dynamic mechanical remodeling of native tissues. Second, p53‐independent mechanisms through which SERPINE1 contributes to senescence, particularly in epithelial cells, remain poorly understood [69]. This limitation also raises the question of whether SERPINE1 exerts similar functions across different cell types and tissue environments or instead acts in a context‐dependent manner shaped by local mechanical and transcriptional states. Although many studies support a pro‐senescent role of SERPINE1, several reports have suggested protective or context‐dependent effects under specific experimental conditions. Therefore, the biological role of SERPINE1 should be interpreted within its specific cellular and mechanical context rather than as uniformly pro‐ or anti‐senescent.

From a therapeutic perspective, several small‐molecule PAI‐1 inhibitors have shown encouraging results in preclinical studies. Tiplaxtinin (PAI‐039) is a representative small‐molecule PAI‐1 antagonist that has demonstrated activity in experimental models [86]. However, clinical translation remains limited by off‐target effects and poor pharmacokinetic properties. Because SERPINE1 also participates in physiological processes such as fibrinolysis and tissue repair, careful consideration of dosing, timing, and tissue specificity will be important for therapeutic application. Combining PAI‐1 inhibitors with senolytics such as dasatinib and quercetin may further reduce senescent cell burden and alleviate fibrosis‐related phenotypes in multiple tissues. Such combination strategies may be particularly effective if SERPINE1 contributes not only to senescence induction but also to the maintenance of a mechanically reinforced senescent microenvironment.

Future studies are needed to further define the role of SERPINE1 in mechanotransduction‐associated senescence. First, clustered regularly interspaced short palindromic repeats (CRISPR) interference (CRISPRi) could be employed to achieve spatiotemporal suppression of SERPINE1 in stiffness‐controlled organoid models, providing validation of the proposed mechanobiological feedback loop. Second, it will be important to determine how ECM stiffness and other mechanical cues influence the phenotypic features of senescent cells and whether the contribution of SERPINE1 differs across these mechanical contexts. Because cellular properties are highly sensitive to the mechanical environment, senescence itself may not represent a uniform state but rather a spectrum of mechanically modulated phenotypes. Clarifying how SERPINE1‐mediated remodeling interacts with these context‐dependent states may provide deeper insight into how local tissue mechanics shape the progression and maintenance of senescence. Third, integrating single‐cell multi‐omics datasets with biomechanical computational models will be valuable for defining context‐dependent functions of SERPINE1 across fibrotic pathologies. Such integrative approaches may also help determine whether SERPINE1 primarily functions as an initiator, amplifier, or stabilizer of senescence under distinct mechanical conditions.

## 7 Conclusion

SERPINE1/PAI‐1 represents a key mechanistic link connecting mechanotransduction, extracellular matrix remodeling, and cellular senescence. Rather than functioning solely as a marker of senescence, SERPINE1 contributes to the persistence of the senescent phenotype through multiple interconnected mechanisms. Mechanistically, SERPINE1 suppresses tPA/uPA‐mediated plasmin generation, limiting ECM degradation while promoting ECM accumulation and matrix stiffening. The resulting stiffened microenvironment further activates integrin‐dependent mechanotransduction pathways, including FAK/Src, RhoA/ROCK, YAP/TAZ, and SMAD signaling, while context‐dependent stress‐responsive pathways may additionally converge on SERPINE1 regulation, thereby contributing to a self‐reinforcing mechanobiological feedback loop.

In parallel, SERPINE1 contributes to a senescence‐associated feedback loop by reinforcing cell‐cycle arrest, maintaining SASP signaling, and promoting profibrotic matrix remodeling. Evidence from fibroblast and epithelial cell contexts suggests that SERPINE1 links intracellular senescence programs with extracellular remodeling that sustains tissue dysfunction. In fibroblasts, SERPINE1 is associated with cytoskeletal remodeling, profibrotic gene expression, and matrix regulation, whereas in epithelial cells, SERPINE1 may promote p53–p21–Rb‐mediated cell‐cycle arrest together with SASP‐driven communication with surrounding stromal cells. These multicellular interactions may be particularly important in fibrotic and age‐associated diseases, where senescent cells, ECM accumulation, and increased tissue stiffness reinforce one another.

Overall, SERPINE1 should be viewed as a context‐dependent mechanobiological mediator that integrates cell‐cycle arrest, inflammatory secretory signaling, and ECM remodeling. Its biological importance is further supported by pharmacological studies using PAI‐1 inhibitors, which suggest that this pathway may represent a potential therapeutic target. However, because SERPINE1 also participates in physiological processes such as fibrinolysis and tissue repair, future studies are needed to define when and where SERPINE1 inhibition may be beneficial. A deeper understanding of SERPINE1‐dependent feedback loops across different cell types and mechanical environments may provide new insight into how senescence is maintained and contributes to chronic tissue remodeling.

## Author Contributions

**Intan Chairun Nisa:** conceptualization, literature review, visualization, writing of the original draft. **Pirawan Chantachotikul:** literature review and scientific discussion. **Kei Takahata:** literature review and scientific discussion. **Arif Nur Muhammad Ansori:** writing of the original draft. **Shinji Deguchi:** conceptualization, supervision, funding acquisition, extensive revision and editing of the manuscript. All authors read and approved the final manuscript.

## Ethics Statement

Ethical approval was not required for this review article because no human participants, animals, or identifiable human data were involved.

## Conflicts of Interest

The authors declare no conflicts of interest.

## Data Availability

Data sharing not applicable to this article as no datasets were generated or analyzed during the current study.
